# Analyzing factors causing deadlock events of bi-directional pedestrian flow when moving on stairs using a personal space model

**DOI:** 10.1038/s41598-024-61007-4

**Published:** 2024-05-13

**Authors:** Mingwei Liu, Guiliang Lu, Oeda Yoshinao

**Affiliations:** 1 Shanghai Ocean University, Shanghai, China; 2 Kyushu University, Kyushu, Japan

**Keywords:** Bi**-**directional movement, Personal space, Deadlock, Pedestrian crowd, Lane formation, Stairs, Civil engineering, Computer science, Risk factors

## Abstract

Comprehending crowd dynamics on staircases is imperative for preventing accidents, particularly in emergency scenarios. In this context, our study delves into bi-directional pedestrian flow. When confronted with limited staircase space, the occurrence of two distinct lanes—one for upstairs and another for downstairs—is a common observation. However, there has been no quantitative investigation conducted to understand this phenomenon. To facilitate such an analysis, we employ a velocity-based personal space model that accurately reproduces the formation of upstairs and downstairs lanes in bi-directional staircases. The study of lane formation mechanisms and the exploration of factors influencing deadlock are essentially two sides of the same coin. This is due to the fact that, the occurrence of deadlock signifies a disruption in the anticipated lane behavior during counter flow. As a result, we have devised various scenarios to meticulously analyze the factors contributing to both deadlock formation and its subsequent performance. This involves manipulating parameters such as speed, speed dispersion, pedestrian count, initial density, right-hand preference weight, minimum personal space size, same-direction following intensity, and time step. The findings hold the potential to enhance the overall quality of service in stairway movement and offer significant contributions to the understanding and management of pedestrian behavior in such settings.

## Introduction

The model of pedestrian traffic flow is relevant for the design and operation of pedestrian facilities as well as for emergency and evacuation planning. The present work exclusively focuses on the modeling of bidirectional flow on straight stairs only.

The term "bidirectional flow", also known as "counter-flow", characterizes the phenomenon in which groups of individuals move in opposing directions while interacting with each other. This occurrence, as demonstrated by Ref.^1^, is quite prevalent. Despite the apparent simplicity of bidirectional setups, they actually illustrate significant mechanisms that lead to the formation of organized lanes. Facilitating the creation of lanes in pedestrian facilities, even under changing circumstances, can significantly mitigate conflicts among pedestrians and enhance their travel speed. This phenomenon of lane formation can be conceptualized as a self-organizing process, wherein pedestrians actively minimize contact with others, particularly when differing walking directions and speeds are involved. Consequently, substantial research attention has been directed towards comprehending bidirectional pedestrian dynamics on level ground in recent decades^2–6^.

Simulation models play an increasingly pivotal role in both the design and assessment of life safety conditions across various domains such as buildings, infrastructure, and outdoor spaces. For instance, numerous modeling approaches have been developed over the years, each grounded in distinct methodologies and assumptions, particularly within the realm of fire safety^7^.

As outlined by Ref.^8^, these models can be categorized into three primary groups: (1) coarse network models, (2) fine network models, and (3) continuous models. These categories signify varying degrees of detail in depicting agent behaviors. Though the coarse network model, which simplifies evacuation representation and may overlook certain evacuation behaviors, has witnessed a decline in adoption by a significant portion of developers and users^9^, two other models have maintained prominence. The continuous model, known as the social force model^10–15^, and the fine network model, referred to as the cellular automata model^16–20^, have emerged as prevalent choices for simulating and analyzing pedestrian dynamics, especially in scenarios involving counter flow.

Considering the subject's significance and scientific interest, numerous laboratory experiments have been conducted to investigate the dynamics of human movement. The experimental data play a crucial role in calibrating and validating models to achieve realistic outcomes. In this context, research^21^ emphasized the importance of microscopic pedestrian simulation models being capable of replicating observed macroscopic fundamental diagrams (FDs) while consistently generating emergent self-organization phenomena at the microscopic level for bidirectional pedestrian flows.

Whether employing the social-force model, the cellular automata model, or other approaches, researchers strive to closely simulate actual pedestrian movement characteristics. While the concept of deadlock, denoting a situation where individuals are immobilized and unable to move, is commonly used in model simulations to represent congestion^22,23^, it rarely occurs in experiments due to even extreme conditions maintaining a minimal flow^24–26^.

However, in the case of two models, deadlock formation was unavoidable, and the simulation's clearance time significantly exceeded that observed in experiments^27^.

Numerous enhanced cellular automata models have been proposed to mitigate deadlock occurrences. These models consider factors such as following behavior^28^, anticipation behavior (evasive effect)^29,30^, dominant row^31^, waiting time rule^27^, and more. Research^32^ highlights a potential cause of deadlock formation: discrete space and time cellular automata models might not accurately replicate the decision-making processes of real individuals. This discrepancy is attributed to the discrete nature of these models, which fail to capture certain real-world adaptive behaviors. Research^32^ proposes several reasons for quantitative disparities between discrete space and time experiments (D-experiments) and conventional experiments: Firstly, pedestrians in D-experiments are rigid, while real pedestrians exhibit flexibility and can adjust their bodies to adapt locally. Secondly, the constraint of one cell per pedestrian in D-experiments limits their movement compared to the flexibility in conventional experiments. Thirdly, the uniform walking speeds in D-experiments contrast with the heterogeneous speeds of pedestrians in conventional situations, where a maximum walking speed corresponds to one cell per step. Consequently, pedestrians in conventional experiments more effectively evade potential conflicts and establish distinct lanes.

Moreover, several enhanced social force models have been developed. For instance, research^33^ introduced the "following effect" and "evasive effect" into their model, primarily contributing to lane formation and reducing the likelihood of deadlock.

Previous research has predominantly focused on flat terrain, with only a limited number of studies addressing the phenomenon of pedestrian counter flow on stairs. A majority of these studies have primarily relied on experiments or observations^34,35^ to derive insights into pedestrian traffic relationships and clearance times, rather than employing simulation models. This preference may stem from the inherent challenges in accurately modeling three-dimensional pedestrian movement using the social-force model or the cellular automata model.

Staircases present an unstable environment due to their vertical orientation, and their capacity is inherently restricted by geometric constraints, leading to the occurrence of "bottleneck" situations. Pedestrian movement on stairs diverges significantly from that on flat surfaces due to several factors:

1. Movement must be differentiated between upward and downward directions.
2. Stair movement entails more physical effort, particularly noticeable among the elderly and children, leading to increased speed disparities. Notably, research^36^ highlighted a broad range of mean local movement speeds during descent, spanning from 0.10  ± 0.008 to 1.7  ± 0.13 m/s.
3. Lane formation tendencies are more pronounced on stairs compared to flat surfaces, a characteristic of pedestrian counter flow dictated by spatial constraints. In narrow staircases, bidirectional flow often results in the formation of two distinct lanes (ascending and descending), irrespective of the distribution of ascending and descending pedestrians^37^.

The research focuses on bidirectional flow on stairs, specifically investigating the underlying mechanisms of deadlock and lane formation. This emphasis arises due to the absence of quantitative descriptions regarding the process of formation or the characteristics of lane creation on staircases. The paper seeks to comprehensively examine the lane formation mechanism and concurrently delve into the factors that influence deadlock. These two aspects are inherently interconnected, representing two facets of the same issue: when pedestrians encounter a deadlock, it signifies a disruption in the expected lane behavior during counter flow.

While it is true that deadlock instances are infrequent or transient in most scenarios, even under competitive conditions, it's important to acknowledge that severe injuries can still occur due to counter flows. In densely populated areas, especially during high pedestrian traffic, such as the 2009 stampede on the Shanghai Bund, pedestrians might find themselves in situations where they cannot easily reverse their direction and move backward, amplifying the risk of accidents.

This study employs a velocity model rooted in the personal space concept, utilizing a first-order microscopic framework to explore pedestrian walking behaviors. This spatially continuous model enables pedestrians to dynamically adjust their speed based on their proximity to the surrounding environment, thus introducing variability into their movement. Should the distance fall below a specific threshold, the speed is reduced to 0, indicating a temporary pause as the pedestrian awaits the distance to surpass the threshold, subsequently resuming movement at a certain pace. In exceptional circumstances, if the threshold-exceeding distance ceases to exist in any forward direction, the pedestrian enters a prolonged deadlock state, remaining stationary. The concept of "following" and "evidence" is incorporated by delineating personal space through varied sizes, creating an egg-shaped zone that reflects different sensitivity levels to the "following" and "evidence" effects. Moreover, the model accounts for anticipation in pedestrian dynamics, wherein individuals anticipate the movements of fellow pedestrians in the upcoming step. This forward-looking behavior prompts pedestrians to yield to oncoming traffic, perceiving a near-future benefit. The model encompasses 21 potential movement directions, allowing individuals to opt for right or left-hand paths, with the intensity of following behavior modifiable through adjustments to model coefficients. Furthermore, if the specifications of the stairs are different, the relationships between personal space size and velocity will differ; therefore, the relationships can reflect the influence of the riser height and the tread width to a certain extent.

Ultimately, the model adeptly captures the intricacies of counter flow on staircases, faithfully replicates individuals' decision-making processes, and offers accurate decision support for emergency response planners and managers^38^.

Our objective is to quantitatively analyze the emergences of deadlocks or lane formations on stairs by investigating the key factors, influencing their occurrence. Additionally, we seek to enhance the realism of pedestrian movement replication. This study explores the effects of five distinct factor categories. The first pertains to the initial pedestrian density and the total pedestrian count. The second category is concerned with speed-related factors, encompassing both speed magnitude and its dispersion. The third category delves into direction selection behavior, encompassing the choice of right-hand movement and the intensity of following behavior. The fourth category delves into the influence of the minimum size of personal space on lane formation. Finally, the fifth category delves into the influence of pedestrian’s reaction time (time step) on lane formation. Through the above categories, pedestrian heterogeneity on lane formation can be taken into account, which is one of the main reasons why doubts still exist on the authenticities of experimental data. In other words, the extent to which such artificial scenarios can represent the intrinsic nature of real-life phenomenon is still questionable^39^.

The outcomes serve a dual purpose: first, to establish the correlation between these factors and the incidence of deadlock, and second, to analyze the attributes of both deadlock and lane formation. This includes aspects such as the commencement time and location of deadlock. The assessment of jamming probability and clearance time serves as the primary metrics for evaluating the simulation models' performance.

The subsequent sections of this paper are structured as follows: "Methods" outlines the concept of PS and elaborates on the development of the PS model used for simulating bidirectional pedestrian flows on stairs. The evaluation of factors impacting deadlock occurrence is detailed in "Results". Lastly, the paper concludes by summarizing the study's findings in the "Conclusions" section.

## Methods

### Concept of personal space (PS)

The basis for the PS (Personal Space) concept, which is utilized to depict interactions and conflicts, is drawn from the work of^40^. An "actual conflict" is delineated as the "physical interruption of or interference with a person's actions or intended actions by other users or the characteristics of the environment, which either blocks a person's behavior or violates their collision zone^41^. It's worth noting that an "actual conflict" doesn't always result in a collision but rather prompts individuals to instinctively avoid contact. This concept of PS is grounded in psychological insights suggesting that humans naturally establish collision zones around themselves, and breaches of these collision zones can lead to discomfort. When faced with potential intrusion, individuals may decide whether to maintain their current movement or adjust it based on their perception of the intrusion's impact.

Furthermore, the influence of other individuals on shared pathways is rooted in human psychology, as people tend to maintain a certain social distance from each other. This behavior reflects a subconscious desire to uphold personal space even in crowded environments.

### Shape of PS

**Figure 1:**
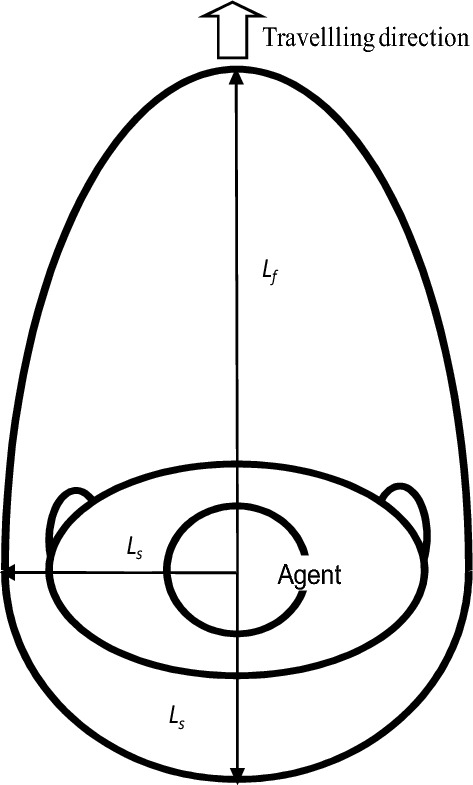
Personal space.

The PS assumes an egg-shaped configuration, as illustrated in Fig. 1. Within this form, the front PS (${L}_{f}$) and the side PS (${L}_{s}$) represent the distances between two individuals in head-on and lateral or rear encounters, respectively. When encountering conflict, individuals typically initiate avoidance behavior, guided by these distances.

**Figure 2:**
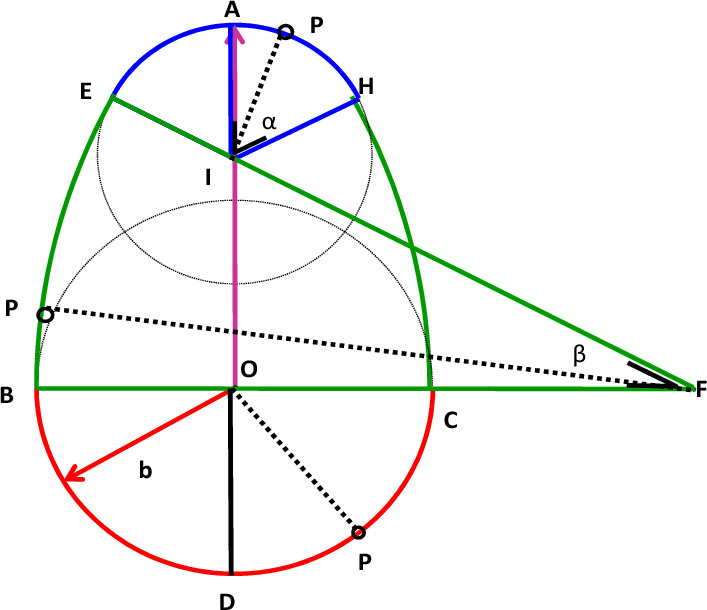
The four arcs that make up a personal space.

This egg-shaped geometry signifies that, compared with lateral and rear movements, the agent distance, that is, the distance people maintain from others to feel comfortable, should be greater in the front movement. In this study, Fig. 2 presents a graphical representation of the PS, featuring four arcs derived from four circles. This illustration showcases the front AS size ($\overline{{\text{OA}} }={L}_{f}$) and the side or rear AS size ($\overline{{\text{OB}} } and \overline{{\text{OD}} }={L}_{s}$), delineated by arcs representing different radii.

Specifically, the figure comprises: Arc 1, a half-circle on the back of the PS centered on the agent with a radius of ${L}_{s}$; Arc 2, situated on the front of the PS centered at I with a radius of *r* and an arc chord angle 2α; Arc 3, centered on F with a radius of *R* and an arc chord angle β; and Arc 4, generated similarly to Arc 3.

This study assumes that the constant proportion of the side or rear length (${L}_{s}$) to the front length (${L}_{f}$) decreases.

### Dynamic characteristics of PS

The dynamic characteristic of PS refers to the relationship between the velocity and size of PS caused by the density of the surrounding agents. When there is a low-density crowd on the stairs, individuals will not be influenced by other pedestrians, and they can walk with the velocity and towards the direction they desire. In this particular free-flow condition, the PS is formed on the basis that the pedestrian hops. As the density increases, his/her activity space decreases, and he/she will reduce his/her speed to maintain a distance from others. To realize friendly movement, we consider that a person should have a comfortable and safe movement function. In general, humans feel uncomfortable and unsafe when their PS is invaded. Thus, if the dynamic PS becomes smaller, a pedestrian will reduce his or her velocity to take shorter strides and maintain a distance from others. This is a favorable solution in narrow environments. Moreover, the decrease in PS and the moving velocity of one person can also cause other pedestrians to feel less oppressed. When the crowd density decreases, more space is available, to relieve discomfort, the PS stretches again, and the velocity increases. Alternatively, in extremely crowded conditions, they are bounded by their body size, and the velocity becomes zero. In summary, these characteristics describe the function of the space headway and the relative velocity between interacting pedestrians *i* and *j*. Hereafter, we will refer to them as the dynamic characteristics of PS. Based on the sizes of the breadth thickness and shoulder width of Chinese people (Table 1), the minimum size of the front PS,${L}_{f}$, and the rear or side PS, ${L}_{s}$ is set to 20 cm.

**Table 1:** Body size for Chinese persons.

	Man (cm)	Woman (cm)
Shoulder width	40.1	37.1
Breadth thickness	21.5	20.8

### Behavior model

**Figure 3:**
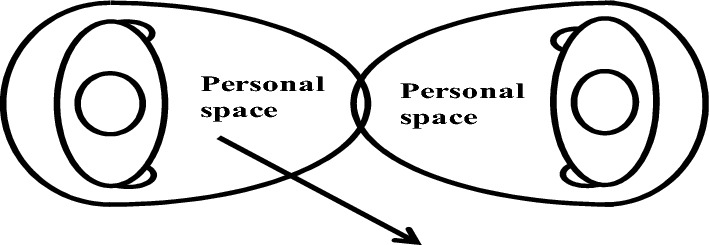
Behavior in conflict avoidance condition.

**Figure 4:**
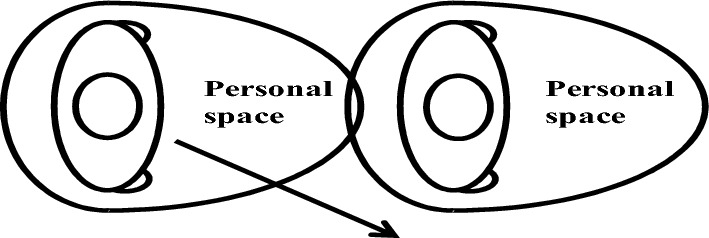
Behavior in overtaking condition.

A behavior model for pedestrians on stairs has been developed, integrating the concept and dimensions of Personal Space (PS), represented by a four-arc shape as depicted in Fig. 2. Figures 3 and 4 further illustrate directional adjustments in scenarios involving conflict avoidance and overtaking, respectively.

In Fig. 3, depicting the conflict avoidance condition where two pedestrians walk in opposite directions, if the PS of the left pedestrian intersects with that of the right pedestrian, the left pedestrian will alter their direction to preserve personal space.

Figure 4 illustrates the overtaking scenario where two pedestrians move in the same direction. Here, if the PS of the left pedestrian comes into contact with that of the right pedestrian, the left pedestrian will adjust their direction accordingly.

In situations with the same direction of movement, pedestrians behind do not necessarily need to maintain personal space like those with opposite directions. However, if a follower's walking speed surpasses that of the pedestrian in front, leading to PS contact, the follower pedestrian will engage in overtaking behavior, as depicted in Fig. 4.

**Figure 5:**
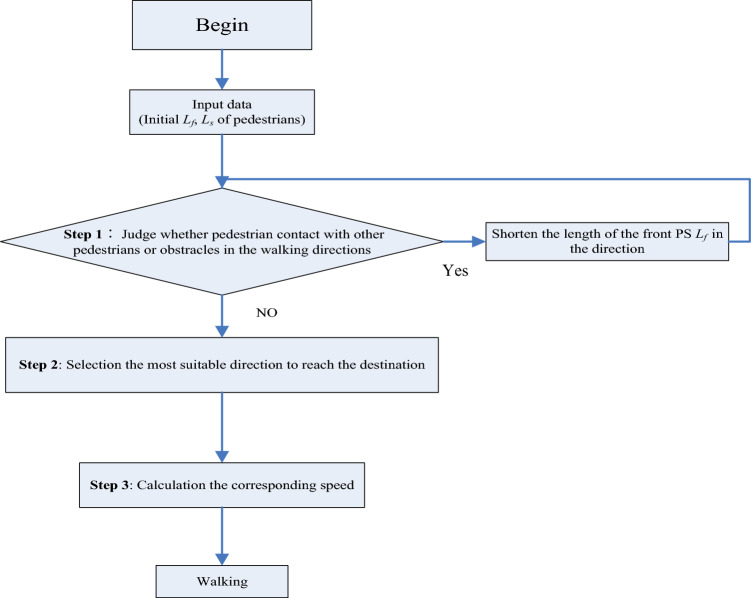
Simulation procedure.

A visual representation of the simulation process is depicted in Fig. 5. The procedure is explained as follows:

Firstly, input the initial size of PS (${L}_{f}$ and ${L}_{s}$) and the number of pedestrians in each side.

**Step 1**: Determine whether a pedestrian comes into contact with other pedestrians or obstacles in all directions. The field spans from − 100° to 100°, totaling 200°, with a scale of 10° as illustrated in Fig. 7. If contact is detected, reduce ${L}_{f}$ in the judged direction by 10 cm and go to step 1 make another judgment, until no contact is found.

**Figure 6:**
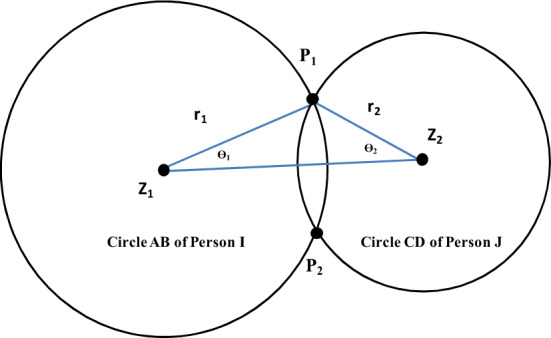
Method used to determine whether circle CD intersects AB.

In our study, the crucial aspect of identifying a PS invasion is dependent on establishing the positional relationship between pedestrians. The method used to determine if the PS of person *I* (*PS*_*I*_) is intruded upon by the PS of person *J* (*PS*_*J*_) involves a two-step process:

Process 1: The initial process involves determining if any of the four circular areas associated with *PS*_*I*_ overlap with any of the four circular areas of *PS*_*J*_. This procedure is depicted in Fig. 6. For instance, Circle AB represents one of *PS*_*I*_'s four circles, centered at *Z*_*1*_ with a radius of *r*_*1*_. Similarly, Circle CD is one of the four circles of *PS*_*J*_, centered at *Z*_*2*_ with a radius of *r*_*2*_. If the condition that ${r}_{1}+{r}_{2}>\overline{{Z }_{1}{Z}_{2}}$ holds true, we can deduce that Circle AB and Circle CD intersect at points *P*_*1*_ and *P*_*2*_. Consequently, utilizing this method allows us to compute the x- and y-coordinates of the contact points *P*_*1*_ and *P*_*2*_ using Eqs. (1)–(7).

1 $$s = \frac{1}{2}\left( {r_{1} + r_{2} + \overline{{z_{1} z_{2} }} } \right)$$

2 $$A = \sqrt {s\left( {s - r_{1} } \right)\left( {s - r_{2} } \right)\left( {s - \overline{{z_{1} z_{2} }} } \right)}$$

3 $$\theta_{1} = \sin^{ - 1} \left( {\frac{2A}{{\left( {\overline{{z_{1} z_{2} }} } \right)\;r_{1} }}} \right)$$

4 $$p_{1x} = z_{1x} + \left( {\cos \theta_{1} \left( {\frac{{z_{2x} - z_{1x} }}{{\overline{{z_{1} z_{2} }} }}} \right) - \sin \theta_{1} \left( {\frac{{z_{2y} - z_{1y} }}{{\overline{{z_{1} z_{2} }} }}} \right)} \right)r_{1}$$

5 $$p_{1y} = z_{1y} + \left( {\sin \theta_{1} \left( {\frac{{z_{2x} - z_{1x} }}{{\overline{{z_{1} z_{2} }} }}} \right) + \cos \theta_{1} \left( {\frac{{z_{2y} - z_{1y} }}{{\overline{{z_{1} z_{2} }} }}} \right)} \right)r_{1}$$

6 $$p_{2x} = z_{1x} + \left( {\cos \left( { - \theta_{1} } \right)\left( {\frac{{z_{2x} - z_{1x} }}{{\overline{{z_{1} z_{2} }} }}} \right) - \sin \left( { - \theta_{1} } \right)\left( {\frac{{z_{2y} - z_{1y} }}{{\overline{{z_{1} z_{2} }} }}} \right)} \right)\;r_{1}$$

7 $$p_{2y} = z_{1y} + \left( {\sin \left( { - \theta_{1} } \right)\left( {\frac{{z_{2x} - z_{1x} }}{{\overline{{z_{1} z_{2} }} }}} \right) + \cos \left( { - \theta_{1} } \right)\left( {\frac{{z_{2y} - z_{1y} }}{{\overline{{z_{1} z_{2} }} }}} \right)} \right)\;r_{1}$$

here, ($${z}_{1x}, {z}_{1y} )$$ are the coordinates of *Z*_1_, ($${z}_{2x}, {z}_{2y}$$) are the coordinates of *Z*_2_, ($${p}_{1x}, {p}_{1y}$$) are the coordinates of contact point *P*_1_, ($${p}_{2x}, {p}_{2y}$$) are the coordinates of contact point *P*_2_, $$\theta_{1}$$ is ∠*P*_1_*Z*_1_*Z*_2_, and $$\theta_{2}$$ is ∠*P*_1_*Z*_2_*Z*_1_.

Process 2: Determine whether intersection point *P*_1_ or *P*_2_ belongs to any one of the four arcs that constitute *PS*_*I*_. The procedure is illustrated in Fig. 2. When ∠*PIA* < ∠*α* (∠*AIH*), intersection point *P* lies on arc *EAH*; when ∠*PFB* < ∠*β* (∠*EFB*), intersection point *P* lies on arc *BE*. When ∠*POD* < 90°, intersection point *P* is on arc *BC*. For arc *HC*, which is derived in a similar manner to arc BE, the method for determining whether *P* belongs to *PS*_*I*_ is similar to that of arc BE. If contact points exist on any arc of *PS*_*I*_, this means the PS of person* I* was invaded.

**Figure 7:**
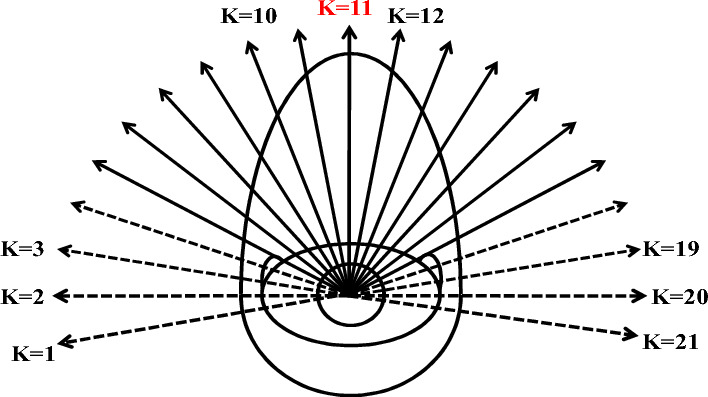
Direction number, *K*

**Step 2** involves selecting the most suitable direction to reach the destination, taking into account the effects of PS size and target direction. The decision-making process for choosing the optimal direction is represented by Eq. (8).

8 $$pro\left(K,t\right)= freq\left(K\right) \times freq\_{L}_{f}(K,t);$$

where $K$ is the direction number (see Fig. 7), $pro\left(K,t\right)$ is the value of choosing direction* K*at time t, the greater this value, the greater likelihood of choosing direction *K*. $pro\left(K,t\right)$ comprises two components.

The first part,$freq\left(K\right)$, represents the influence of the target destination. The values of $freq\left(K\right)$ are listed in Table 2. Direction numbers range from 1 to 21, with $K$ = 11 representing the head-on direction. Directions from 1 to 10 are on the left-hand side of the head-on direction, while those from 12 to 21 are on the right-hand side. The maximum value of $freq(K)$ is associated with *K* = 11, and values decrease as the direction deviates from this central direction. Figure 7 displays two types of direction arrows: solid and dotted. Solid arrows denote directions within the field of vision, spanning from − 60° to 60°, totaling 120°. Dotted arrows indicate outside directions, ranging from − 100° to − 70° and from 70° to 100°. Notably, the values of $freq\left(K\right)$ within the field of vision do not exhibit bilateral symmetry; those on the right are slightly higher due to the prevalence of right-handed individuals favoring rightward choices. Values in the field of vision (*K* = 5–17) are notably higher compared to those outside.

**Table 2:** Values of $freq(K)$^40^.

*K* = 1	*K* = 2	*K* = 3	*K* = 4	*K* = 5	*K* = 6	*K* = 7	*K* = 8	*K* = 9	*K* = 10	*K* = 11
0.0001	0.0053	0.0131	0.0292	1.00003	1.00004	1.00005	1.00006	1.00007	1.00008	1.00009

*K* = 12	*K* = 13	*K* = 14	*K* = 15	*K* = 16	*K* = 17	*K* = 18	*K* = 19	*K* = 20	*K* = 21	
1.000085	1.000075	1.000065	1.000055	1.000045	1.000035	0.0292	0.0131	0.0053	0.0001	

The second part, $freq\_{L}_{f}(K,t)$, accounts for the effect of the front PS in direction *K* at time *t*. For pedestrians moving in opposite direction, $freq\_{L}_{f}(K,t)$ is described by Eq. (9). Conversely, for pedestrians moving in the same direction, $freq\_{L}_{f}(K,t)$ is described by Eq. (10).

Opposite direction:

9 $$fre{q}_{{L}_{f}\left(K,t\right)}= \frac{{L}_{f}\left(K ,t\right)}{100};$$

Same direction:

10 $$\begin{gathered} {\text{When}}\,\,\,L_{f} \left( {K,t } \right) < 150\,\,{\text{cm}},\,\,\,freq_{{L_{f} \left( {K,t} \right)}} = \frac{{L_{f} \left( {K,t } \right)}}{50}; \hfill \\ {\text{When}}\,\,L_{f} \left( {K,t } \right) \ge 150\,\,{\text{cm}},\,\,\,\,freq\_L_{f} \left( {K,t} \right) = \frac{aa\left( K \right)}{{100}}, \hfill \\ \end{gathered}$$

${\text{where}}, {L}_{f}(K,t)$ (cm) is the front PSin direction $K$ at time *t*, $aa\left(K\right)$(cm) is the initial front PS in direction $K$, which is a normal distribution with an average value of 290 cm and a deviation of 0.45.

The formulation in Eqs. (9) and (10) reflects the reality where the tendency to follow pedestrians in the same direction outweighs that of walking in opposite directions, leading to reduced conflict.

The direction in which the pedestrian will take their next step is determined by the maximum value of $pro\left(K,t\right)$, which integrates the weight equations for destination orientation and the size of the front PS.

In** Step 3**, once the optimal direction is chosen, the pedestrian proceeds to determine their subsequent walking step. This movement is influenced by both the selected direction and the walking speed. The pace of movement depends on the size of the frontal PS (${L}_{f}$) in the chosen direction, as described in Eqs. (11) and (12). The relationship between the front PS, ${L}_{f}$(cm), and ascending velocity ${V}_{up}$(cm/s) is expressed in Eq. (11).

11 $$\begin{gathered} {\text{When}}\,\,L_{f} < 110;\,\, V_{up} \in \left\{ {{ }0.4582{ }L_{f} { } + { }8.3921{ } - { }9.06,{ }0.4582L_{f} { } + { }8.3921{ } + { }9.06{ }} \right\}; \hfill \\ {\text{When 11}}0 \, \le L_{f} < 290;\,\, V_{up} { } \in \left\{ {{ }0.0489L_{f} { } + { }59.282{ } - { }13.64,{ }0.0489L_{f} { } + { }59.282{ } + { }13.64{ }} \right\}; \hfill \\ {\text{When}}\,\,L_{f} \ge 290;\,\, V_{up} \in \left\{ {{ }0.0101L_{f} + 73.741 - 14.17,{ }0.0101L_{f} + 73.741 + 14.17{ }} \right\}. \hfill \\ \end{gathered}$$

The relationship between the front PS, *L*_*f*_ (cm), and descending velocity ${V}_{down}$ (cm/s) is expressed as Eq. (12).

12 $$\begin{gathered} {\text{When}}\,L_{f} < 120;\,\,V_{down} \in \left\{ {{ }0.4625L_{f} { } + { }11.156 - 9.38,{ }0.4625L_{f} { } + { }11.156 + 9.38{ }} \right\}; \hfill \\ {\text{When}}\,\,120\,\, \le L_{f} < 290;\,\, V_{down} \in \left\{ {{ }0.0971L_{f} { } + { }57.331 - 14.6,{ }0.0971L_{f} { } + { }57.331 + 14.6{ }} \right\}; \hfill \\ {\text{When}}\,\,L_{f} \ge 290;\,\,V_{down} \in \left\{ {{ }0.0021L_{f} { } + { }86.383 - 18.7,{ }0.021L_{f} { } + { }86.383 + 18.7{ }} \right\}. \hfill \\ \end{gathered}$$

**Figure 8:**
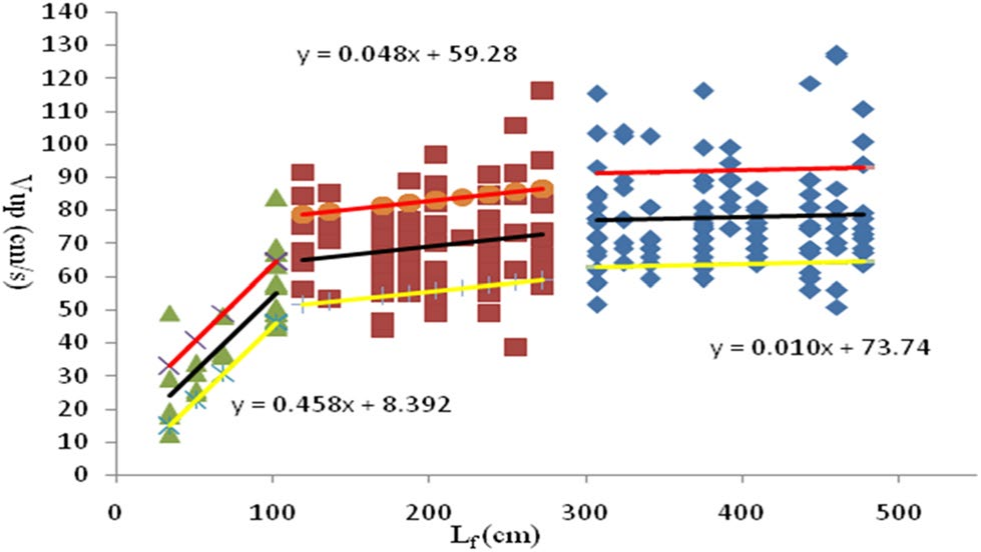
Relationship between the ascending speed ${V}_{up}$ and ${L}_{f}$.

**Figure 9:**
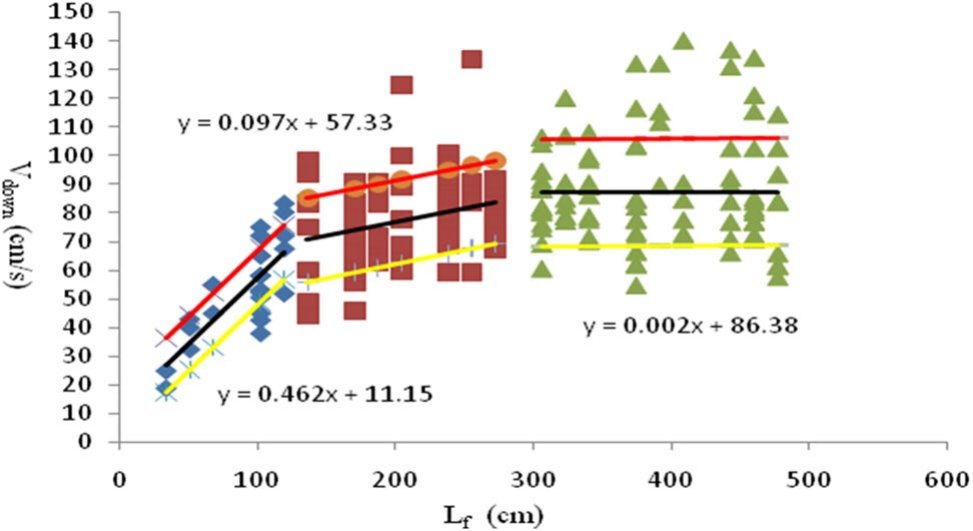
Relationship between the descending speed ${V}_{down}$ and ${L}_{f}$.

The speed range is defined by the red and yellow lines in Figs. 8 and 9. This range covers 80% of the recorded survey data. The black line, positioned between the red and yellow lines, represents the line-fitting curves obtained from the original dataset, as described in reference^38^.

For the next step, the distance traveled by the pedestrian equals their speed in the chosen direction multiplied by the time step.

## Results

**Figure 10:**
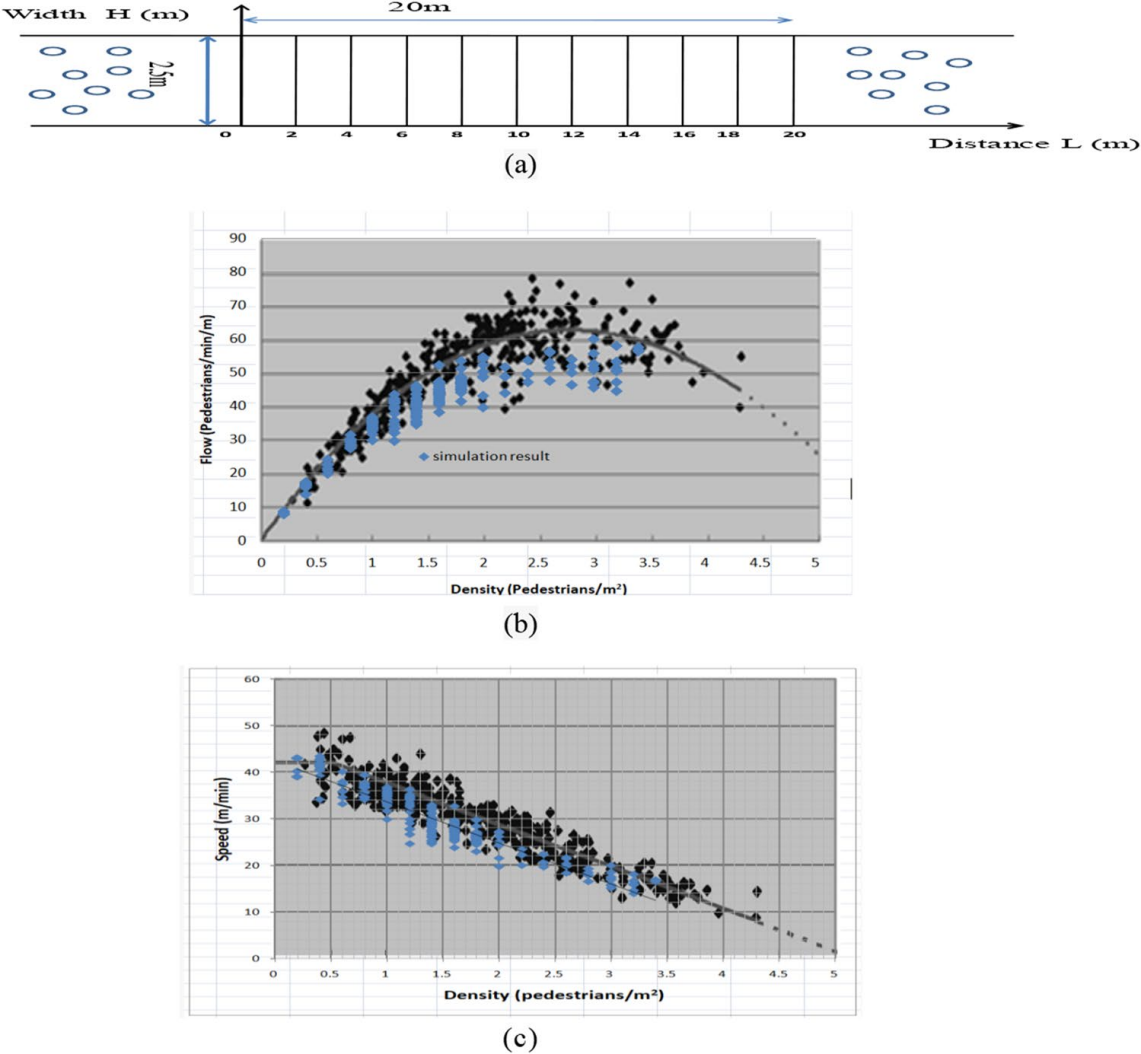
(**a**) Staircase; (**b**) comparison of the simulation result and the results from research^34^ (flow-density relationship). (**c**) Comparison of the simulation result and the results form research^34^ (speed-density relationship).

A series of simulations were conducted to explore the factors influencing the occurrence and behavior of deadlock situations. As depicted in Fig. 10a, the staircase is 2.5 m wide and 20 m long, with a time step of 0.5 s. The number of ascending (84) and descending (84) pedestrians is equal, totaling 168 pedestrians.

Previous studies have successfully used walking experiments and observations to calibrate and validate pedestrian simulation models^42–45^and interpret various crowd phenomena like self-organization^44^ and lane formation^46^. To validate our model, we referred to the research by^34^, which studied pedestrian flow on two-way straight stairways in Shanghai Metro stations. Figure 10b,c show the comparison between our simulation results for the (2,100) combination and the findings of research^34^. While our model's results slightly underestimate the actual flow-density and speed-density relationships, they remain largely consistent. This indicates that our model accurately reflects daily stair walking behavior through precise parameter settings. Building on this, future studies will refine parameters and analyze factors affecting deadlock phenomena.

### Definition of different states

**Figure 11:**
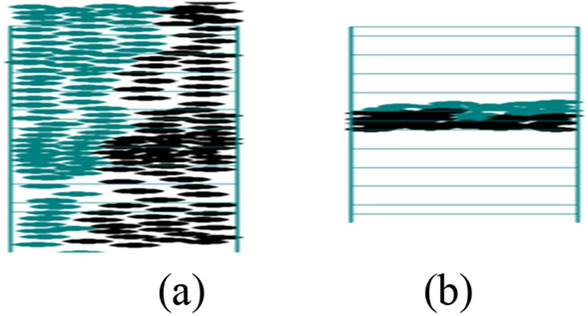
Definition of patterns. (**a**) Lanes. (**b**) Deadlock.

Two significant patterns identified within the system modeling counter flow on stairs are lanes and deadlocks, each elucidated as follows (refer to Fig. 11):

1. Lane: In this pattern, nearly every column of cells exclusively accommodates one type of pedestrian, with only minor deviations due to fluctuations. The staircase is divided into two lanes, specifically for ascending and descending pedestrians (as illustrated in Fig. 11a).
2. Deadlock: This term refers to total congestion within the system, where upstairs and downstairs pedestrians obstruct each other to such an extent that any progress in the intended direction becomes impossible, ultimately resulting in a velocity of zero (depicted in Fig. 11b).

#### Meaning of nomenclature

The simulation outcomes include deadlock probability, deadlock start time, and clearance time, defined as follows:

- Deadlock Probability: This metric represents the likelihood of a specific configuration leading to a complete halt in pedestrian flow. To ensure reliability, each simulation was iterated 20 times, and the deadlock probability was calculated by dividing the count of deadlock events by the total number of executions. A higher value indicates a greater likelihood of deadlock occurrence, reflecting increased obstruction in the flow process.
- Deadlock Start Time (s): This parameter denotes the moment when a pedestrian first stops moving on the stairs during a deadlock event. The average deadlock start time is calculated as the mean value across all deadlock trials. A larger value indicates a later occurrence of deadlock. This parameter to some extent reflects the level of chaos and disorder. Generally, the more chaotic and disorder, the faster the deadlock occurs.
- Clearance Time (s): This metric represents the time required for all pedestrians to exit the stairs. In cases where deadlock-forming simulations cannot continue, clearance time is only counted for passing trials. The average clearance time is obtained by averaging all successful passing trials. A shorter average clearance time indicates faster evacuation of all pedestrians from the stairs and smoother pedestrian flow.

### Factors affecting deadlock probability

This section explores the factors influencing the likelihood of encountering a deadlock. We analyze various potential psychological and physiological factors, including:

1. Initial density
2. Weight assigned to selecting the right-hand direction
3. Speed
4. Intensity of following the same direction
5. Diversity in speeds
6. Pedestrian count
7. Minimum personal space size
8. Time step

The outcomes of these analyses are summarized in the subsequent section.

#### Case A: Result from analyzing initial density

In this simulation, all settings remain consistent with those outlined in "Methods", except for changes in the initial density. The initial density is determined by the number of pedestrians in each line (PN) and the distance between each line (DL) in centimeters. A higher PN value indicates a higher density, while a shorter DL value results in a higher density. There are 16 combinations of PN and DL, with their corresponding initial densities listed in Table 3. In this table, the first number in each combination represents PN, while the second number represents DL. The initial density is calculated using Eq. (13).

13 $${\text{Iinitial Density}} = \frac{{2{\text{PN}}}}{{2.5\left( {\frac{{{\text{DL}}}}{100}} \right)}}$$

**Table 3:** The combination of PN and DL and the initial density.

Combinations of PN and DL	2, 100	3, 100	4, 100	6, 100	2, 70	3, 70	4, 70	6, 70	2, 50	3, 50	4, 50	6, 50	2, 40	3, 40	4, 40	6, 40
Initial density (pedestrians/m^2^)	1.6	2.4	3.2	4.8	2.3	3.4	4.6	6.9	3.2	4.8	6.4	9.6	4	6	8	12

**Figure 12:**
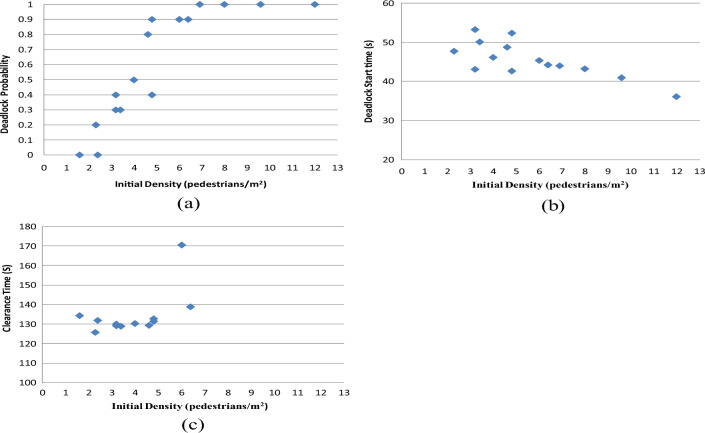
(**a**) Relationship between the initial density and the deadlock probability; (**b**) relationship between the deadlock start time and the initial density; (**c**) relationship between the clearance time and the initial density.

For example, the combination of (2, 100) indicates that the pedestrian number in every line is two, the head-on distance between every line is 100 cm, and the initial density is 1.6 pedestrians/m^2^. In the 16 combinations, (4, 100) and (2, 50), as well as (6, 100) and (3, 50), have the same density. Both combinations for different numbers of PN and DL are listed in Table 3. The outcomes are displayed in Fig. 12. The discoveries are outlined as follows:

The probability of deadlock occurrence escalates in direct proportion to the initial density, as illustrated in Fig. 12a. Similarly, maintaining a constant row space, a higher number of individuals per line corresponds to an increased probability of deadlock. Furthermore, keeping the pedestrian count constant in each line while decreasing the row space also leads to a higher probability of deadlock. Therefore, mitigating the initial density has the potential to lower the likelihood of deadlock.

Deadlock start time decreases as the initial density increases, as depicted in Fig. 12b. Notably, lower initial densities result in a more varied range of deadlock start times. This occurs because as the initial density increases, the degree of chaos also increases, leading to quicker deadlock occurrences.

The clearance time initially diminishes and then rises with an increase in the initial density, as shown in Fig. 12c. When the initial density exceeds 5 pedestrians/m^2^, the clearance time increases sharply. This effect arises because at lower initial densities, pedestrian positions are more spaced out, resulting in longer clearance times. As the initial density increases, the clearance time initially decreases until reaching its minimum. However, continued growth in the initial density results in heightened pedestrian interactions, leading to increased congestion levels and consequently, longer clearance times.

#### Case B: Results from analyzing right-hand selection weight

This section conducted two distinct simulation scenarios to assess the impact of weighting associated with right-hand selections on deadlock probability. For these simulations, only the variable $freq\left(K\right)$ is modified, while other parameters remain consistent with those detailed in "Methods".

CASE B1: The frequencies ($freq\left(K\right)$) for right-hand directions (*K* ranging from 11 to 21, as shown in Fig. 7) are set at twice the values indicated in Table 2.

CASE B2: The frequencies ($freq\left(K\right)$) for right-hand directions (*K* from 12 to 17) are equal to the frequencies of the corresponding left-hand directions (*K* from 10 to 5).For example,$freq\left(17\right)$=$freq\left(5\right)$=1.00003.

**Figure 13:**
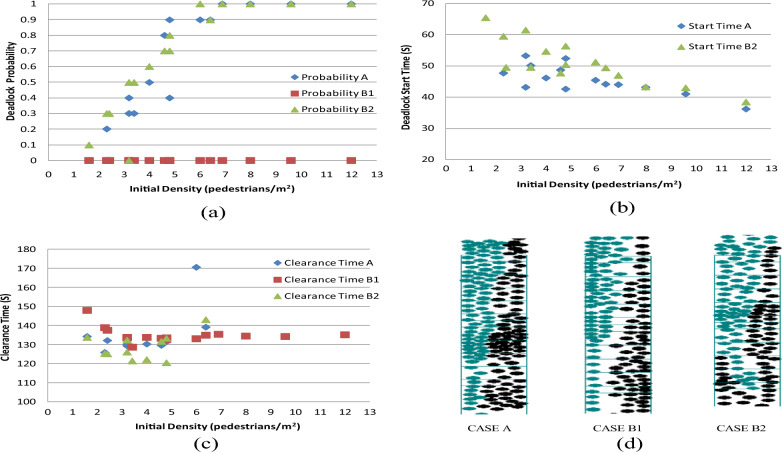
(**a**) Comparison of the deadlock probability; (**b**) comparison of the deadlock start time; (**c**) comparison of the clearance time; (**d**) comparison of the simulation snapshot in the fifth trial of (2,50) combination.

Figure 13a–c illustrates the comparative findings for deadlock probability, deadlock start time, and clearance time across varying initial densities. Figure 13d provides a visual snapshot of the simulation.

The comparative outcomes are outlined below:

Enhancing the weight attributed to the right-hand direction choice substantially diminishes the deadlock probability, regardless of the initial density (Fig. 13a). This highlights that establishing a more consistent walking pattern can notably reduce the incidence of deadlocks. Additionally, the formation of lanes is generally considered to counteract deadlock occurrences. Conversely, in CASE B2, the deadlock probability surpasses that of CASE A. This discrepancy arises due to a reduction in the extent of direction selection regularity, leading to an elevated likelihood of deadlock occurrence.

The deadlock start time in CASE B2 is slightly larger than that in CASE A (Fig. 13b). This may be because when the corresponding right and left hand directions are equal, there are more directions to choose from than in CASE A, which delays the occurrence of deadlocks. Moreover, both cases exhibit a pattern of decreasing with increasing initial density.

Clearance times for both CASE A and CASE B2 follow a trend of initial reduction followed by eventual increase as initial density grows. Furthermore, the values exhibit more variability than those observed in CASE B1 (Fig. 13c). In CASE B2, when the initial density is low or medium, equal selection probabilities for corresponding left and right directions can reduce the clearance time. In the context of CASE B1, clearance time experiences an initial decrease, and when the initial density surpasses 3 pedestrians/m^2^, the value converges to approximately 134 s.

CASE B1 and CASE A manifest themselves as two queues, both upstairs and downstairs (Fig. 13d). When compared with CASE A, the separation between the queues in CASE B1 is more pronounced. This increased distance between the queues on different floors reduces interactions between opposing face-to-face movements, likely contributing to the reduction in deadlock probability. In contrast, despite sharing identical initial conditions with CASE A, CASE B2 gives rise to three queues due to equal selection probabilities for left and right directions. This increased complexity in movement pattern makes the occurrence of deadlock more likely.

#### Case C: Results from analyzing speed

This section investigated the influence of speed on the likelihood of encountering deadlocks, as well as its impact on deadlock start time and clearance time. The investigation encompassed two simulated scenarios:

CASE C1: The speed of both upstairs and downstairs movements was increased to 1.5 times the original speed.

CASE C2: The speed of upstairs and downstairs movements was set at 0.9 times the original speed within the lower half of the area delimited by the black and yellow lines (as depicted in Figs. 8 and 9).

**Figure 14:**
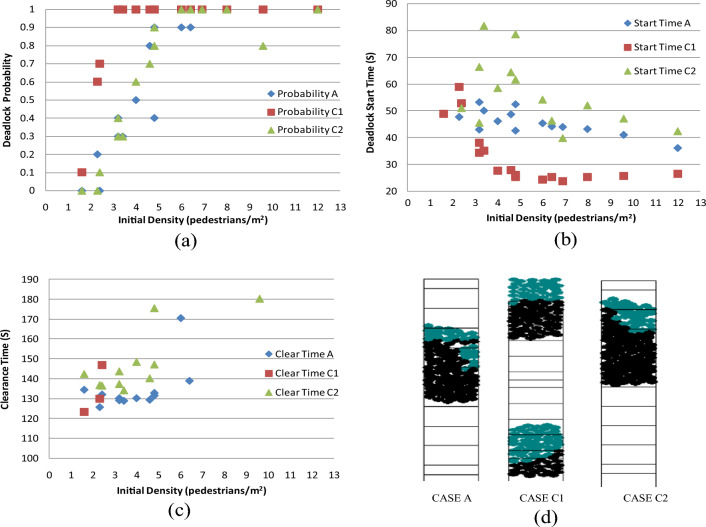
(**a**) Comparison of the deadlock probability; (**b**) comparison of the deadlock start time; (**c**) comparison of the clearance time; (**d**) comparison of the deadlock position from the first trial in (3,40) combination.

Comparative analyses of deadlock probability, deadlock start time, and clearance time are presented in Fig. 14a–c, respectively. Furthermore, Fig. 14d illustrates a comparison of deadlock positions among the three scenarios under the first trial in (3, 40) combination.

The observed outcomes are outlined as follows:

Augmenting the speed noticeably heightens the probability of deadlock, while reducing the speed still does not reduce the deadlock probability (Fig. 14a). It's worth noting that in this study, the speed was only decreased by a modest 10%. However, a substantial decrease in speed can lead to severely restricted pedestrian mobility, especially when initial density is high. This mobility constraint results in pedestrians remaining stationary at the beginning of the simulation due to minimal spacing and slow speed. Although this might also amplify the deadlock probability, a compromise was reached to maintain pedestrian mobility by setting the speed at 90% of the original value for the lower half (between the black and yellow lines).

Faster speeds expedite the initiation of deadlocks, while reduced speeds extend the time before deadlock initiation (Fig. 14b). Substantially lowering the speed significantly prolongs the clearance time. Interpretation of results in CASE C1 was difficult due to the limited availability of only three data points (Fig. 14c), indicating that only under low initial density conditions, passing events can be observed in CASE C1.

Upon increasing the speed by 1.5 times, the deadlock occurrence shifts to the lower and upper ends of the staircase (Fig. 14d). In contrast, in CASE A and CASE C2, the deadlock remains situated on the stairs themselves. These findings underscore that when pedestrians ascend and descend rapidly, there is a heightened risk of obstructing the lower and upper staircase areas, potentially resulting in accidents.

#### Case D: Result from analyzing same direction following intensity

This section explores the influence of the intensity of same-direction following. Two distinct simulation scenarios were executed:

CASE D1: Enhancement of same-direction following intensity, accomplished by adopting a direction choice probability as per Eq. (14), which is twice the magnitude of Eq. (10).

Same direction:

14 $$\begin{gathered} {\text{When}}\,\,L_{f} \left( {K,t } \right) < 150\,\,{\text{cm}},\,\,pro\left( {K,t} \right) = \frac{{L_{f} \left( {K ,t} \right)}}{25} \times freq\left( K \right); \hfill \\ {\text{When}}\,L_{f} \left( {K,t } \right) \ge 150\,\,{\text{cm}},\,\,pro\left( {K,t} \right) = { }\frac{aa\left( K \right)}{{50}} \times freq\left( K \right), \hfill \\ \end{gathered}$$

CASE D2: Reduction in the intensity of same-direction following, resulting in a direction choice probability as defined by Eq. (15), which is the same with that of the opposite direction (Eq. (9)).

Same direction:

15 $$\begin{gathered} {\text{When}}\,\,L_{f} \left( {K,t } \right) < 150\,\,{\text{cm}},\,\,pro\left( {K,t} \right) = \frac{{L_{f} \left( {K ,t} \right)}}{100} \times freq\left( K \right); \hfill \\ {\text{When}}\,\,L_{f} \left( {K,t } \right) \ge 150\,\,{\text{cm}},\,\, pro\left( {K,t} \right) = \frac{{L_{f} \left( {K ,t} \right)}}{100} \times freq\left( K \right). \hfill \\ \end{gathered}$$

**Figure 15:**
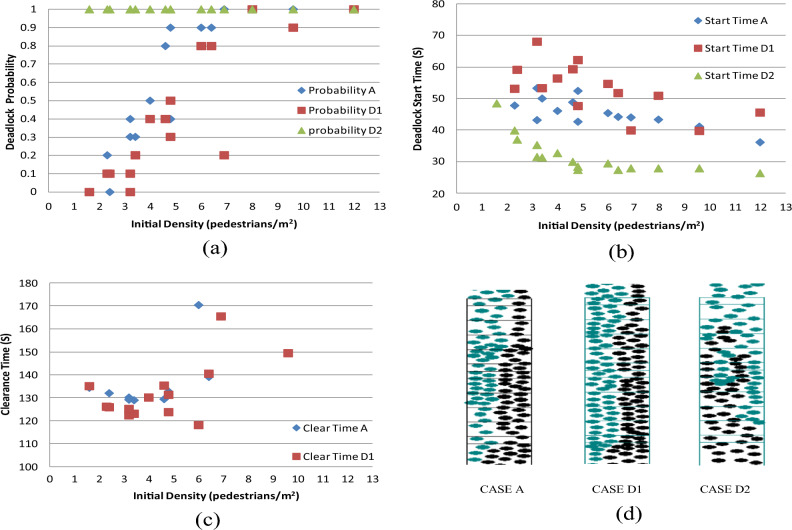
(**a**) Comparative analysis of deadlock probability; (**b**) comparative analysis of deadlock start time; (**c**) comparative analysis of clearance time; (**d**) comparative snapshot of simulation in the third trial of the (2,40) Combination.

Comparative analyses of deadlock probability, deadlock start time, and clearance time are depicted in Fig. 15a–c, while Fig. 15d provides a visual snapshot of the simulation.

The outcomes of these simulations are detailed below:

Increasing the probability of same-direction following can lead to a certain reduction in deadlock probability, although the impact is not as pronounced as seen in CASE B1.This enhancement promotes a more cohesive flow pattern, reducing the likelihood of conflicting movements that can result in deadlocks. Conversely, decreasing this intensity significantly elevates the likelihood of deadlock (Fig. 15a).

Deadlock start times tend to occur earlier in scenarios with reduced same-direction following intensity and vice versa (Fig. 15b). This suggests that while weaker same-direction following behavior may accelerate the onset of congestion.

The trend in clearance time for both CASE A and CASE D1 is similar, wherein an initial decrease is followed by an increase as density rises. Although the distinction between the two cases is minimal, the clearance time in CASE D1 is smaller than that in CASE A, which means increasing following intensity can slightly decrease the clearance time. Yet, the initial density associated with CASE D1 is higher than that of CASE A, indicating that even under high initial density conditions, passing events are still observed in CASE D1 (Fig. 15c).

The simulation snapshots demonstrate that CASE A and CASE D1 exhibit two distinct columns for bidirectional movement upstairs and downstairs. However, CASE D2 displays interspersed gaps and a greater number of columns, contributing to an increased likelihood of deadlock occurrence (Fig. 15d). This phenomenon also illustrates that overtaking occurs less frequently as the intensity of following in the same direction increases, thus leading to the formation of two queues: one upstairs and one downstairs. If the intensity of following decreases, overtaking becomes more likely, resulting in multiple queues as pedestrians behind change direction to catch up with those ahead.

#### Case E: Results from analyzing speed diversity

CASE E involves two types of simulations:

CASE E1 entails broadening the speed dispersion range by a factor of four. As a result, the linear correlations between the front PS,${L}_{f}$ (cm), and the upward velocity, ${V}_{up}$ (cm/s), can be expressed using Eq. (16).

16 $$\begin{gathered} {\text{When}}\,\,\,L_{f} < 110:\,\,V_{up} \in \left\{ {{ }0.4582{ }L_{f} { } + { }8.3921{ } - { }4 \times 9.06,{ }0.4582L_{f} { } + 8.3921{ } + { }4{ } \times { }9.06{ }} \right\}; \hfill \\ {\text{When}}\,\,110 \le L_{f} < 290:\,\,V_{up} \in \left\{ {{ }0.0489L_{f} + 59.282{-}{ }4 \times 13.64,{ }0.0489L_{f} { }59.282{ } + { }4 \times 13.64{ }} \right\}; \hfill \\ {\text{When}}\,\,L_{f} \ge 290:\,\,\,V_{up} \in \left\{ {{ }0.0101L_{f} { } + { }73.741{ }{-}{ }4{ } \times { }14.17,{ }0.0101L_{f} { } + { }73.741{ } + { }4{ } \times { }14.17{ }} \right\}. \hfill \\ \end{gathered}$$

The relationships between the front PS, $L_{f}$ (cm), and descending velocity,$V_{down}$(cm/s),are defined by Eq. (17).

17 $$\begin{gathered} {\text{When}}\,\,L_{f} < 120:\,\,V_{down} \in \left\{ {{ }0.4625L_{f} { } + { }11.156 - 4 \times 9.38,{ }0.4625L_{f} { } + { }11.156 + 4 \times 9.38} \right\}; \hfill \\ {\text{When}}\,\,{12}0 \le L_{f} < 290:\,\,V_{down} \in \left\{ {{ }0.0971L_{f} { } + { }57.331 - 4 \times 14.6,{ }0.0971L_{f} { } + { }57.331 + 4 \times 14.6} \right\}; \hfill \\ {\text{When}}\,\,L_{f} \ge 290:\,\,V_{down} \in \left\{ {{ }0.0021L_{f} { } + { }86.383 - 4 \times 18.7,{ }0.021L_{f} { } + { }86.383 + 4 \times 18.7} \right\}. \hfill \\ \end{gathered}$$

CASE E2: Omitting the speed dispersion component from Eqs. (11) and (12). Consequently, the relationship between the front pedestrian space, $L_{f}$ (cm), and ascending velocity, $V_{up}$ (cm/s), follows a linear form as defined in Eq. (18).

18 $$\begin{gathered} {\text{When}}\,L_{f} < 110:\,\,\,V_{up} = 0.4582{ }L_{f} { } + { }8.3921; \hfill \\ {\text{When}}\,\,110 \le L_{f} < 290:\,\,V_{up} = 0.0489L_{f} { } + { }59.282; \hfill \\ {\text{When}}\,L_{f} \ge 290:\,V_{up} = 0.0101L_{f} { } + { }73.741. \hfill \\ \end{gathered}$$

The linear correlations between the front pedestrian space, ${L}_{f}$(cm), and the descending velocity ${V}_{down}$(cm/s) are defined by Eq. (19).

19 $$\begin{gathered} {\text{When}}\,\,{ }L_{f} < 120:\,\,V_{down} = 0.4625L_{f} { } + { }11.156; \hfill \\ {\text{When}}\,\,120\,\, \le L_{f} < 290:\,\,V_{down} = 0.0971L_{f} { } + { }57.331; \hfill \\ {\text{When}}\,L_{f} \ge 290:\,\,V_{down} = 0.0021L_{f} { } + { }86.383. \hfill \\ \end{gathered}$$

**Figure 16:**
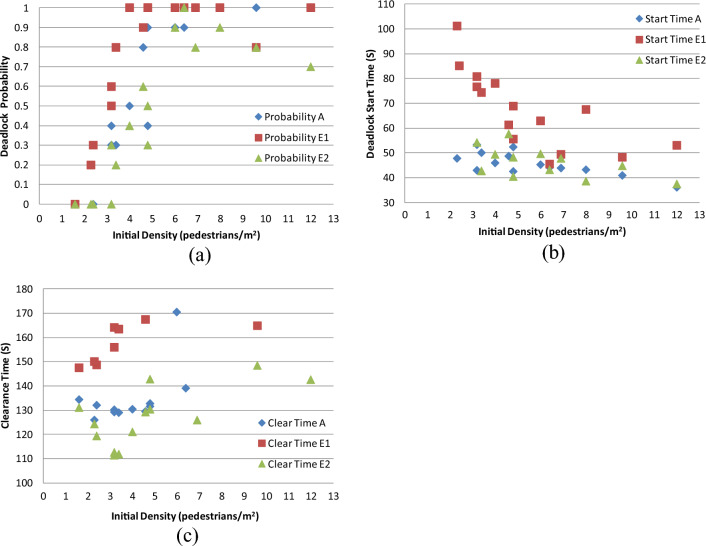
(**a**) Comparative analysis of deadlock probability; (**b**) comparative analysis of deadlock start time; (**c**) comparative analysis of clearance time.

Figure 16a–c present the comparative findings. The outcomes derived from the speed dispersion analysis reveal the following trends:

Elevating speed dispersion correlates with an increased likelihood of deadlock, while reducing speed dispersion demonstrates the opposite effect (Fig. 16a). Notably, even under conditions of high initial density, a reduction in speed dispersion can mitigate the probability of deadlock occurrence.

Increasing speed dispersion obviously extends the deadlock start time (Fig. 16b).

Increasing speed dispersion corresponds to a noticeable increase in clearance time, and vice versa (Fig. 16c).

#### Case F: Results from analyzing pedestrian number

This section encompasses two distinct simulation scenarios:

CASE F1: The pedestrian count is elevated to 168 upstairs and 168 downstairs movements, totally 336 pedestrians.

CASE F2: The pedestrian count is reduced to 48 upstairs and 48 downstairs, totally 96 pedestrians.

**Figure 17:**
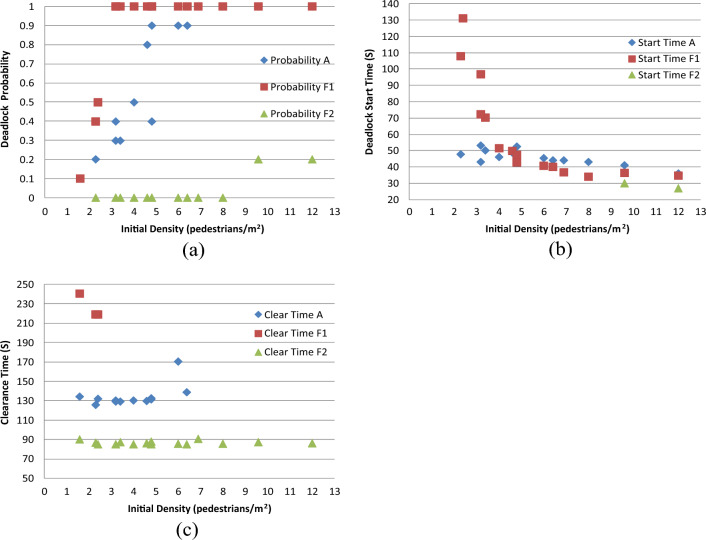
(**a**) Comparative analysis of deadlock probability; (**b**) comparative analysis of deadlock start time; (**c**) comparative analysis of clearance time.

Figure 17a–c provide comparative analyses for deadlock probability, deadlock start time, and clearance time, respectively.

The conclusions can be summarized as follows:

Increasing the number of pedestrians leads to a significant increase in the probability of deadlock occurrence, and conversely, reducing the number of pedestrians decreases this probability (Fig. 17a). This finding underscores the connection between deadlock occurrence probability and congestion duration. Longer periods of congestion correspond to higher probabilities of deadlock. Notably, even in CASE F1, under the (2, 100) combination, the deadlock probability reaches 0.1. This demonstrates that lowering the initial density remains an effective strategy to mitigate the probability of deadlock occurrence, even with high pedestrian numbers.

When the initial density is less than 4 pedestrians/m^2^, the deadlock occurrence time of CASE F1 is much higher than that of CASE A. However, when the initial density exceeds this value, the disparities in deadlock start times among the three cases are marginal, and the value in CASE F1 becomes slightly smaller than that in CASE A. However, the limited sample size of CASE F2 prevents a definitive determination of the temporal trend based on initial density (Fig. 17b).

Analysis of clearance time reveals that in CASE F1, the value is notably greater than that in CASE A, while in CASE F2, it is notably smaller. The disparities in clearance time among various pedestrian numbers are evident. Interestingly, in CASE F2, the distinction in clearance time is minimal, indicating that the initial density has a minor impact on clearance time when pedestrian numbers are low (Fig. 17c).

#### Case G: Results from analyzing smallest dimension of Personal Space Size

In this section, two distinct simulation scenarios were undertaken:

CASE G1: Minimizing the smallest dimensions of ${L}_{f}$ and ${L}_{s}$ to 10 cm.

CASE G2: Expanding the smallest dimensions of ${L}_{f}$ and ${L}_{s}$ to 30 cm. Due to the interplay between the smallest dimension of personal space size and the width of the stairs, only six combinations ((2,100), (3,100), (4,100), (2,70), (3,70), and (4,70)) were conducted in CASE G2.

**Figure 18:**
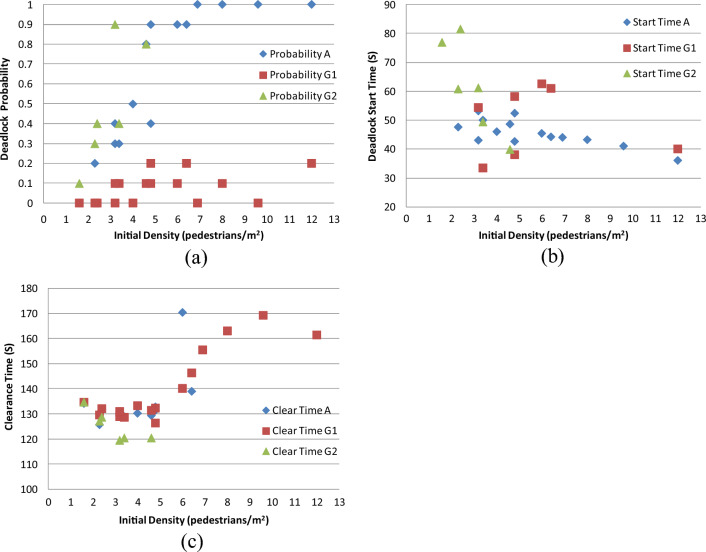
(**a**) Comparative analysis of deadlock probability; (**b**) comparative analysis of deadlock start time; (**c**) comparative analysis of clearance time.

Figure 18a–c offer comparative analyses for deadlock probability, deadlock start time, and clearance time, respectively.

The conclusions can be summarized as follows:

A reduction in the minimum personal space size leads to a substantial decrease in deadlock probability, and vice versa (Fig. 18a). This reduction in size diminishes the hindrance to counter flow and consequently lowers the occurrence of deadlocks.

Decreasing the minimum personal space size results in a more scattered distribution of deadlock start times. In CASE G2, as the density increases, the deadlock start time quickly decreases (Fig. 18b).

Reducing the minimum personal space size leads to a slight increase in clearance time, and vice versa (Fig. 18c). The reason is that a larger personal space size means faster speed.

#### Case H: Results from analyzing time step

In this section, two distinct simulation scenarios were undertaken:

CASE H1: Minimizing the time step to 0.2 s.

CASE H2: Expanding the time step to 0.8 s.

**Figure 19:**
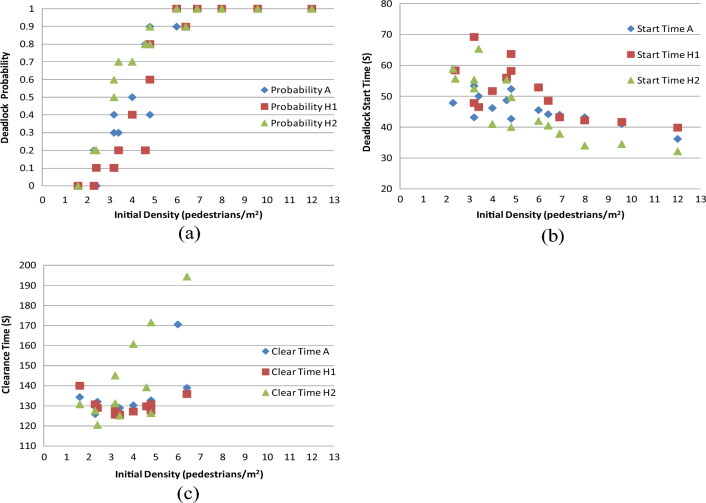
(**a**) Comparative analysis of deadlock probability; (**b**) comparative analysis of deadlock start time; (**c**) comparative analysis of clearance time.

Figure 19a–c offer comparative analyses for deadlock probability, deadlock start time, and clearance time, respectively. The conclusions derived from the analysis of the time step can be summarized as follows:

Decreasing the time step slightly reduces the probability of encountering deadlocks, while increasing the time step leads to a slight increase in deadlock probability (Fig. 19a). The ability to quickly identify and adjust to the positions of pedestrians moving in the opposite direction plays a crucial role in reducing conflicts and consequently lowering the likelihood of deadlocks.

A decrease in the time step results in a slight increase in deadlock start time, while increasing the time step leads to a slight decrease in deadlock start time (Fig. 19b). This observation suggests that a finer time resolution allows for more accurate and timely adjustments in pedestrian movement, delaying the onset of deadlock events.

Increasing the time step introduces greater dispersion in clearance time, while decreasing the time step reduces this dispersion (Fig. 19c). However, it's noteworthy that the differences in clearance time between CASE A and CASE H1 are minimal, indicating that the impact of the time step on clearance time variability may not be significant compared to other factors analyzed.

### Findings from the comparison with study^35^

**Figure 20:**
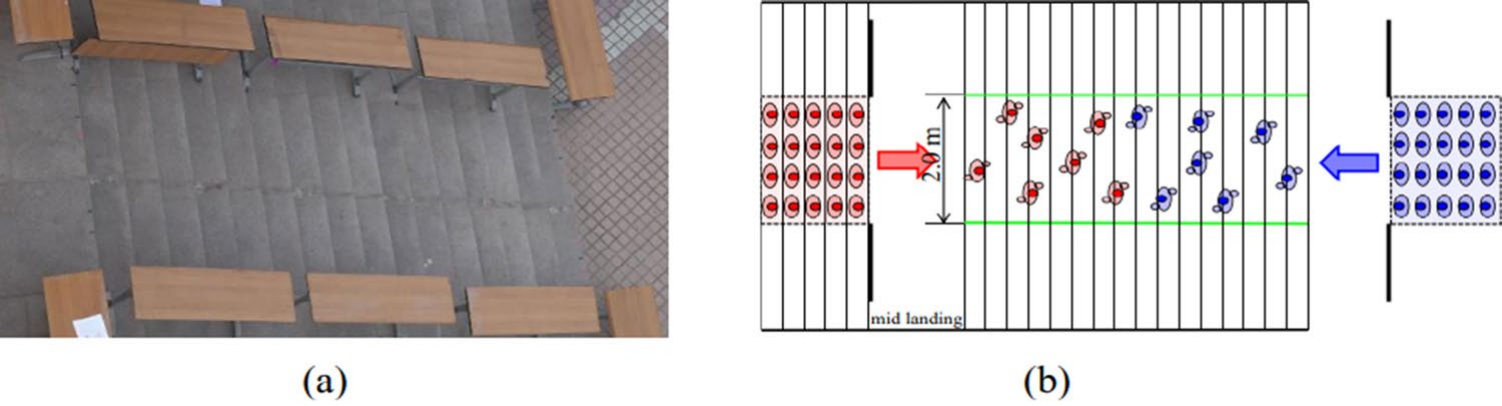
Illustration of the experiment setup. (**a**) Snapshot; (**b**) sketch^35^.

To validate our findings, we referred to the study conducted by research^35^, which involved bi-directional experiments on a staircase with 100 students. Figure 20 illustrates the experiment setup through a snapshot (a) and a sketch (b). The staircase's dimensions are 2 m in width and 5 m in length. Their research indicated that deadlock never occurred, and the highest density reached 7 persons/m^2^.

To facilitate comparison, we conducted simulations that took into account factors influencing lane formation. The objective of the simulation was to obtain crossing speeds and a fundamental diagram while maintaining the same staircase dimensions as those in research^35^. The simulation scenario encompassed a combination of (4, 100), where the downstairs ratios were set as (0.2, 0.3, 0.4, 0.5, 0.6, 0.7, and 0.8) as in^35^. The downstairs ratio is defined as the ratio of downstairs pedestrians to the total number of pedestrians. Mid landings of 1.2 m were positioned on both sides, placing the initial positions of upstairs and downstairs pedestrians 1.2 m away from the stairs. Two simulation cases were hold under following conditions.

In Case 1, the parameter settings were as follows: (1) The minimum ${L}_{f}$ and *L*_*s*_ of each pedestrian were set to 10 cm. (2) The right-hand selection weight was set to match CASE B1. (3) No speed dispersion was introduced, and the relationships between front personal space and speed followed Eqs. (18)–(19). (4) The intensity of same-direction following was established according to Eq. (14). (5) The time step was set to 0.2 s.

Case 2 was conducted with each pedestrian's minimum ${L}_{f}$ and *L*_*s*_ were set to 20 cm, while keeping the other settings consistent with those mentioned in points (2)–(5) above.

For each ratio, we conducted five trials. In CASE 1, deadlock did not occur in any of the trials. However, in CASE 2, deadlock was observed in trials corresponding to ratios 0.2, 0.3, 0.4, 0.6 and 0.7. The frequency of deadlock occurrence is detailed in Table 4. The findings provide additional evidence that the minimum size of ${L}_{f}$ and *L*_*s*_ has a significant impact on the occurrence of deadlock.

**Table 4:** Deadlock occurrence number in CASE 2.

Downstairs ratio	0.8	0.7	0.6	0.5	0.4	0.3	0.2
Deadlock occurrence number	0	1	2	0	1	2	2

**Figure 21:**
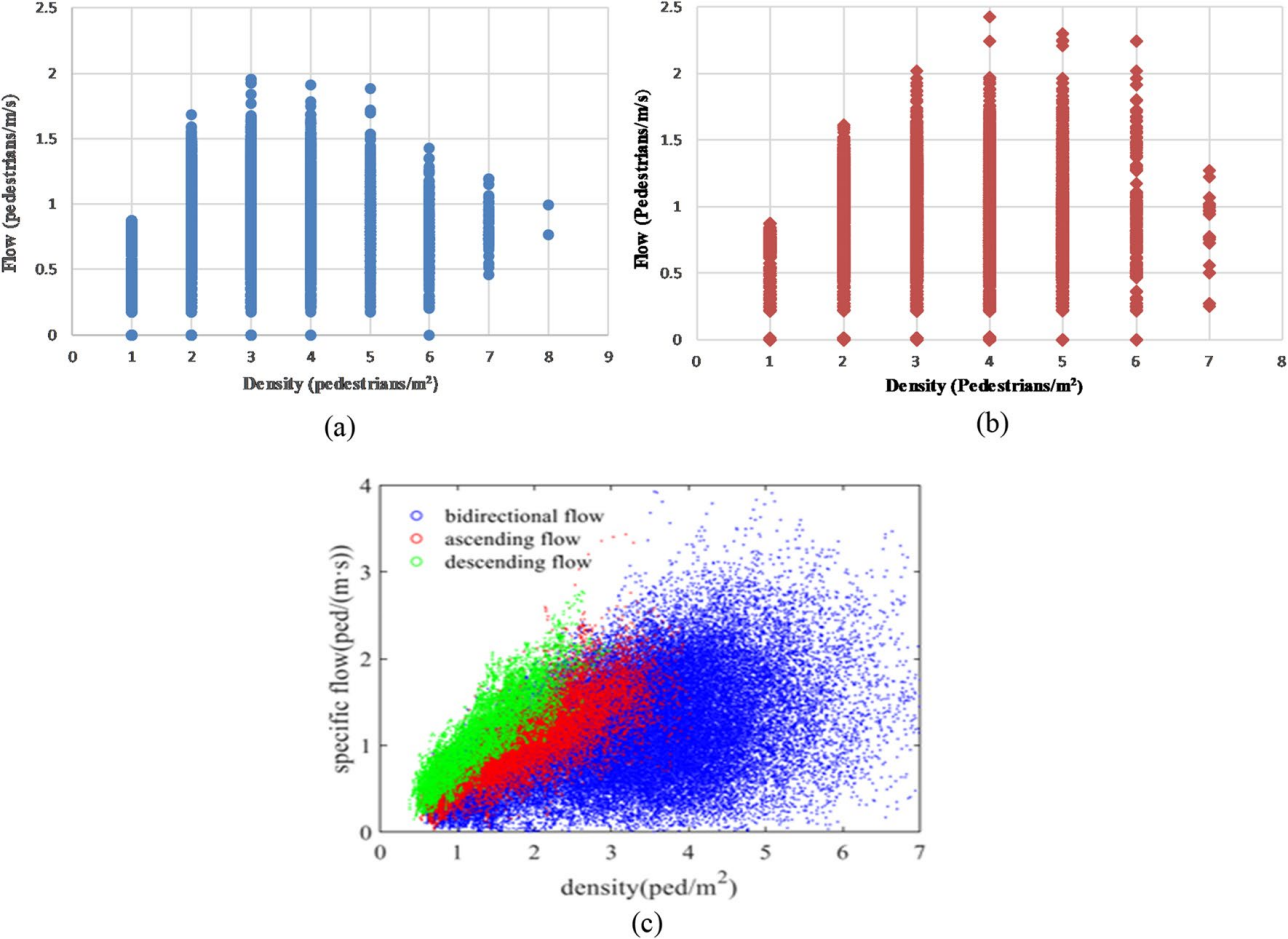
Fundamental diagrams on stairs. (**a**) Simulation results (minimum ${L}_{f}$ and *L*_*s*_ = 10 cm); (**b**) simulation results (minimum ${L}_{f}$ and *L*_*s*_ = 20 cm); (**c**) scatter plot (research^35^).

The average crossing speeds of pedestrians moving in both upward and downward directions for the 0.5 ratio are presented in Tables 5 and 6. Table 5 displays the results from CASE 1, while Table 6 displays the results from CASE 2. In every trial, the mean was computed from data gathered from 50 pedestrians ascending and 50 pedestrians descending. This computation entailed dividing 500 cm by the crossing time, which denotes the duration a pedestrian required to move from entering to departing the stairs. The fundamental diagrams illustrating the simulation results from Case 1 and Case 2 are depicted in Fig. 21a,b, respectively. Additionally, Fig. 21c presents a scatter plot based on the findings of research^35^.

**Table 5:** The case 1 and experiment results of crossing speed (50 upstairs and 50 downstairs).

	Trial 1	Trial 2	Trial 3	Trial 4	Trial 5	Average value of 5 trials	Result of research^35^
Average upstairs crossing speed (cm/s)	27	25.0	29.4	37.6	29.7	30	34
Average downstairs crossing speed (cm/s)	30.2	29.2	37.8	27.6	34.3	32	50

**Table 6:** The Case 2 and experiment results of crossing speed (50 upstairs and 50 downstairs).

	Trial 1	Trial 2	Trial 3	Trial 4	Trial 5	Average value of 5 trials	Result of research^35^
Average upstairs crossing speed (cm/s)	28.4	23.2	25.6	18.3	29.3	25	34
Average downstairs crossing speed (cm/s)	25.0	26.9	26.8	24.3	34.3	27.5	50

Key findings:

The simulation revealed a maximum density of 8 pedestrians/m^2^ in Case 1 and 7 pedestrians/m^2^ in Case 2. In Case 1, this density is slightly higher than what that reported in research^35^, while in Case 2, it matches the reported density. It's noteworthy that the peak bi-directional flow observed in research^35^ nearly reached 4 pedestrians/m/s (Fig. 21c), while the simulations yielded values of 1.96 pedestrians/m/s (Fig. 21a) and 2.426 pedestrians/m/s (Fig. 21b) in Case 1 and Case 2, respectively. The maximum flow in research^35^ surpasses that in Cases 1 and 2. This difference may arise from variations in velocity, as supported by the crossing speed results (Tables 5 and 6). More precisely, in research^35^, the average speeds are 34 cm/s (upstairs) and 50 cm/s (downstairs), whereas in Case 1, they are 30 cm/s (upstairs) and 32 cm/s (downstairs), and in Case 2, they are 25 cm/s (upstairs) and 27.5 cm/s (downstairs). The average speed in CASE 2 is slower than in CASE 1. This could be attributed to the increase in minimum personal space size leading to higher congestion levels and consequently reduced speeds.

From the perspective of deadlock occurrence and speed, the results of CASE 1 and research^35^ are closer. However, compared to research^35^, the simulation results show lower speeds, both in the upstairs and downstairs directions. In CASE 1, the upstairs speed is only slightly lower than the upstairs speed in research^35^. However, the downstairs speed in research^35^ is much higher than the downstairs speeds in CASE 1.Furthermore, the simulation indicates similar values for upstairs and downstairs speeds when the downstairs ratio is 0.5, whereas the experiment exhibits a noticeable disparity with the two kinds of speed. This difference may arise from pedestrians' greater adaptability to adjust their speeds in real-world experiments. Despite variations in downstairs speed, it is noteworthy that deadlock did not occur in either the simulation CASE 1 or the experiment. The maximum density observed also showed similarity among the three contexts. Moreover, the factors discussed earlier exhibit a discernible impact on deadlock occurrence to a certain extent.

## Conclusions

This study presents a novel velocity-based microscopic model that utilizes the concept of personal space to analyze bidirectional movement on staircases. The model not only investigates factors influencing the emergence of lane formations or deadlocks but also provides insights for enhancing pedestrian safety on stairs. By manipulating parameters such as speed, speed dispersion, pedestrian count, initial density, right-hand preference weight, minimum personal space size, same-direction following intensity, and time step, various scenarios were examined to identify contributors to deadlock occurrences. The investigation included detailed analyses of deadlock start times, deadlock probabilities, and clearance times, contributing to a deeper understanding of pedestrian dynamics within lane formations on bi-directional stairs and laying theoretical groundwork for effective pedestrian flow management.

To validate our findings, we conducted simulations involving the aforementioned factors and compared the outcomes with other research. The experimental and simulation results show discrepancies in speed and maximum flow. Furthermore, deadlock occurred in the simulation results when the minimum sizes of ${L}_{f}$ and *L*_*s*_ were 20 cm. Future investigations should focus on refining the model in terms of directional and speed selections to enhance its congruence with real-world scenarios (Supplementary Information S1).

### Supplementary Information

Supplementary Information.

## Data Availability

All data generated or analyzed during this study are included in this published article [and its supplementary information files].
